# Synthesis and Properties of Silk Fibroin/Konjac Glucomannan Blend Beads

**DOI:** 10.3390/polym10080923

**Published:** 2018-08-18

**Authors:** Carla Giometti França, Vicente Franco Nascimento, Jacobo Hernandez-Montelongo, Daisy Machado, Marcelo Lancellotti, Marisa Masumi Beppu

**Affiliations:** 1 Faculdade de Engenharia Química, Universidade Estadual de Campinas, Campinas 13083-852, São Paulo, Brazil; carlagfranca@gmail.com (C.G.F.); vicente.qmc@gmail.com (V.F.N.); beppu@feq.unicamp.br (M.M.B.); 2 Departamento de Ciencias Matemáticas y Físicas, Facultad de Ingeniería, Universidad Católica de Temuco, Temuco 4813302, Chile; 3 Núcleo de Investigación en Bioproductos y Materiales Avanzados (BioMa), Facultad de Ingeniería, Universidad Católica de Temuco, Temuco 4781312, Chile; 4 Laboratório de Biotecnologia, Instituto de Biologia, Universidade Estadual de Campinas, Campinas 13083-862, São Paulo, Brazil; daisy.machado@gmail.com (D.M.); mlancell@unicamp.br (M.L.)

**Keywords:** biopolymers, silk fibroin, konjac glucomannan, porous beads, scaffolds, tissue engineering

## Abstract

Silk fibroin (SF) and konjac glucomannan (KGM) are promising materials in the biomedical field due to their low toxicity, biocompatibility, biodegradability and low immune response. Beads of these natural polymers are interesting scaffolds for biomedical applications, but their fabrication is a challenge due to their low stability and the necessary adaptation of their chemical and mechanical properties to be successfully applied. In that sense, this study aimed to synthesize a blend of silk fibroin and konjac glucomannan (SF/KGM) in the form of porous beads obtained through dripping into liquid nitrogen, with a post-treatment using ethanol. Intermolecular hydrogen bonds promoted the integration of SF and KGM. Treated beads showed higher porous size, crystallinity, and stability than untreated beads. Characterization analyses by Fourier-transform infrared spectroscopy (FTIR), thermogravimetric (TGA), and X-ray diffraction (XDR) evidenced that ethanol treatment allows a conformational transition from silk I to silk II in SF and an increase in the KGM deacetylation. Those chemical changes significantly enhanced the mechanical resistance of SF/KGM beads in comparison to pure SF and KGM beads. Moreover, samples showed cytocompatibility with HaCaT and BALB/c 3T3 cells.

## 1. Introduction

Global challenges in scaffolds technology offer opportunities for further research that involves new types of polymers in different forms such as films, membranes, and beads. In the last decades, natural polymers have been investigated to be used as cell scaffolds [1]. However, to be successfully used these materials should be modified to improve different properties, such as in-vivo stability, biocompatibility, biodegradability, and potential interaction with cells. For that, the scaffolds should also have a cell-friendly surface, and interconnected pores for the transport of cells, nutrients, and metabolites [2].

One of the most promising materials to be used as cell scaffolds, among other applications in biomedicine is silk fibroin (SF), which is a biodegradable polymer, and biocompatible with human tissues. Its chemical composition consists of simple amino acids, such as glycine, alanine, serine, and tyrosine [3,4]. SF is a molecule with high tensile strength, elasticity, and resilience. It has been used in diverse applications, such as hydrogels synthesis, supports for enzymes immobilization, drug delivery, and etc. [3,5,6]. Konjac glucomannan (KGM) is also a biodegradable and biocompatible material [1]. Its chemical structure is composed of d-mannose and d-glucose monomers linked by β(1→4) bonds. KGM is a hydrophilic polymer with high viscoelasticity and mechanical resistance. Over the last years, it was mainly used as a bioactive polymer. KGM, combined with other polymers, can be used as a controlled delivery system for drugs, and antimicrobial materials [7,8].

In spite of the mentioned properties of SF and KGM, their mechanical properties could be improved in order to be used as a scaffold. Blending two or more polymers is a usual technique to overcome challenges in polymer properties and resulting in the new materials. Several studies of SF-based blends have been reported in the literature. Park et al. (1999) verified that the mechanical tensile strength of SF and chitosan (CHI) membranes depended on CHI concentration. Moreover, the resistance of membranes presented higher value than pure polymers, due to the increase in β-sheet conformational transition [9]. Kweon et al. (2001) studied films of SF and CHI to understand the effect of fibroin and chitosan ratio on the physical characteristics of blends. They verified that the mechanical properties the films were improved when blends were formed with 10–40% chitosan [10]. Lee et al. (2004) studied the physical and structural properties of SF and sodium alginate blends and concluded that the structural characteristics of SF in blends were not affected by the incorporation of sodium alginate. Also, the mechanical properties of these blends were improved because sodium alginate is a hard polysaccharide [11]. She et al. (2008) reported that the mechanical properties of SF/CHI scaffolds could be controlled by adjusting their composition [12].

In that sense, this study was focused on the synthesizes and characterization of porous blend beads obtained from two natural polymers, SF and KGM, with suitable characteristics to be applied to tissue engineering or drug release field [13]. A surface morphological analysis was performed by scanning electron microscope (SEM), physicochemical characterizations were obtained by Fourier-transform infrared spectroscopy (FTIR), thermogravimetric (TGA), and X-ray diffraction (XDR), and a validation of mechanical resistance was performed using compression tests. Finally, cytocompatibility assays were carried out using two kinds of cell lines: human keratinocytes (HaCaT) and murine fibroblast (BALB/c 3T3).

## 2. Materials and Methods

### 2.1. Beads Synthesis

#### 2.1.1. Solutions Preparation

Bombyx mori silkworm cocoons were supplied by Fiação Bratac (Bastos-SP, Brazil). Cocoons were degummed three times by soaking in Na_2_CO_3_ 1 g/L solution at 85 °C for 30 min. Afterward, the obtained SF fibers were rinsed with distilled water to remove Na_2_CO_3_ residues and dried at room temperature (25 °C) for 24 h. A ternary solvent of CaCl_2_:ethanol:H_2_O in a molar ratio of 1:2:8 was used to dissolve the SF fibers to 10% (*w*/*v*) at 85 °C for 1.5 h. The SF salt solution was dialyzed by a cellulose membrane (MWCO 3500, Viscofan®, Spain) against distilled water for 72 h at 4 °C, with changes every 24 h [5,14]. The obtained concentration of SF solution was 5% (*w*/*v*), which was determined by casting the solution in a petri dish and weighing the dried mass after solvent evaporation. Finally, the dialyzed solution was diluted in deionized water to a concentration of 2.5% (*w*/*v*).

KGM (Konjac Glucomannan Powder®, Sunnyvale, CA, USA) was dissolved in deionized water to a concentration of 1 g/L. After homogenization, Ca(OH)_2_ (Synth^®^, Diadema, Brazil) was added at a concentration of 1.5 mmol/L to pre-stabilize KGM [15,16,17]. Calcium was used to crosslink the KGM, to make it insoluble in water.

The blends were prepared by adding the solution of SF 2.5% (*w*/*v*) into KGM 1% (*w*/*v*) at a weight ratio of 50% (*w*/*w*) under continuous stirring, and this maintained for 5 min for complete homogenization.

#### 2.1.2. Beads Synthesis

**Figure 1:**
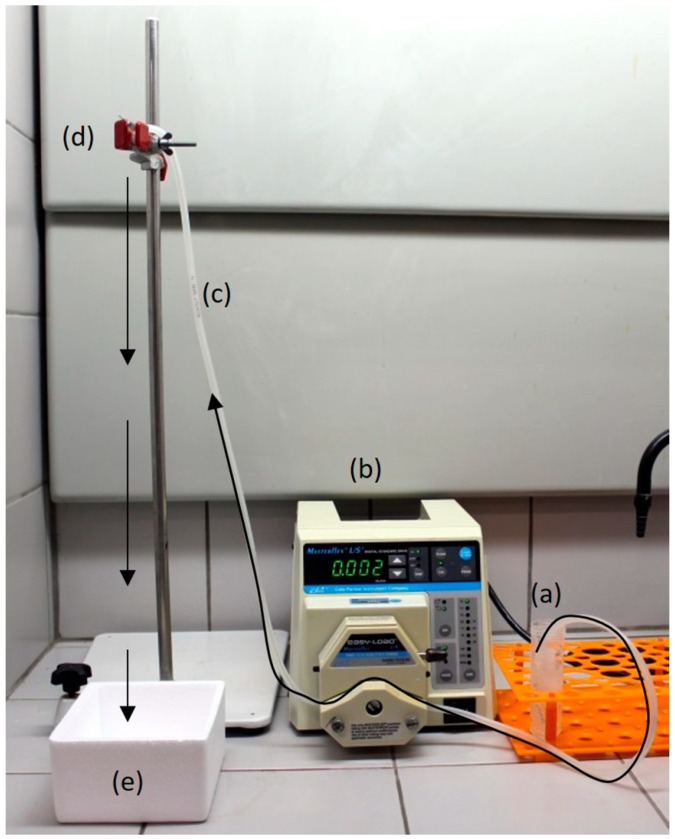
Experimental setup for beads syntheses: (**a**) natural polymer solution; (**b**) peristaltic pump; (**c**) silicone hos; (**d**) needle and (**e**) liquid nitrogen.

SF, KGM, and SF/KGM beads were prepared by using a peristaltic pump (7518-00, Masterflex^®^, Gelsenkirchen, Germany) engaged in a silicone hose (diameter: 0.3 mm, Masterflex^®^, Gelsenkirchen, Germany) and a needle (diameter: 0.70 mm × 30 mm, BD SoloMed, Curitiba, Brazil). Each solution was pumped to the needle and dripping into liquid nitrogen (fast freezing) for the formation of beads. Figure 1 shows the used experimental setup. Finally, the obtained beads were freeze-dried (L101, Liobras, São Carlos, Brazil) for 24 h.

To study the effect of a post-treatment with ethanol, SF, KGM, and SF/KGM beads were treated with ethanol 95% (*v*/*v*) at room temperature for 24 h. Ethanol was used to induce SF structural transformation and KGM deacetylation [15,16,17]. After treatment, beads were rinsed with deionized water, frozen at 4 °C and lyophilized for 24 h.

### 2.2. Physicochemical Characterizations

#### 2.2.1. Surface Tension and Size Beads

The surface tension of solutions was determined by a tensiometer (K12, KRUSS, Hamburg, Germany) using the ring method [18]. The size of beads was obtained analyzing fifteen images of each kind of sample using Image J^®^ software (National Institutes of Health, Bethesda, USA).

#### 2.2.2. Morphological Characterization

The morphology of the surface beads was examined using a scanning electron microscope (FEG 250, Thermo Scientific Quanta™, Waltham, MA, USA) with a current and voltage of 50 pA and 10 kV, respectively. The average pore size of the beads was determined from SEM images by ImageJ® software.

The samples were fixed on aluminum supports using double-sided carbon tape, and in sequence, the samples were metallized using a 200 Å thick gold layer. Metallization was performed in a vacuum chamber (K-450, Sputter Coater EMITECH, Quorum technologies, Lewes, UK) under argon flow for 15 min. After metallization was completed, the supports containing the metalized samples were placed under a microscope, and the samples were photographed for recording purposes.

#### 2.2.3. Chemical Characterization

Attenuated total reflection Fourier-transform infrared spectroscopy (ATR-FTIR) was used for the chemical characterization of the samples. Analyses were carried out with an ATR-FTIR-6100 (JASCO, Easton, MD, USA) spectrometer. Each measurement was acquired in transmittance mode by an accumulation of 128 scans at a resolution of 4 cm^−1^ in the range of 650–4000 cm^−1^. Deconvolution of FTIR spectra was obtained by a Gaussian function using the Fityk 0.9.8 software (Marcin Wojdyr, Warsaw, Poland). The percentage of β-sheet conformation was evaluated by integrating the curve area of the whole studied region.

#### 2.2.4. Thermogravimetric Characterization

Thermogravimetric characterization was performed by TGA equipment (TGA/DSC1, Mettler Toledo, Barueri, Brazil) in a temperature range of 25–600 °C with a ramp rate of 10 °C/min, and an N_2_ flow of 50 mL/min. The glass transition temperature (*T*_g_) was obtained by a differential scanning calorimeter (DSC1, Mettler-Toledo, Barueri, Brazil). All measurements were carried out from 25 to 300 °C under a nitrogen atmosphere using a heating rate of 10 °C/min.

#### 2.2.5. Crystallinity

The crystallinity of beads was studied by X-ray diffraction (X’Pert-MPD, Philips panalytical X-ray, Malvern Panalytical, Almelo, The Netherlands) with Cu-Kα radiation (λ = 1.54056 Å). The X-ray source was operated at 40 kV and 40 mA. The measurements were performed in a range of 2θ = 5–80° using a scanning speed of 0.033°/s with a step size of 0.02°.

### 2.3. Mechanical Assays

The mechanical properties of freeze-dried beads were measured by confined compression test using a TA.XT2 texturometer (Stable Micro Systems, Godalming, UK) with a 50 N load cell at room temperature. The speed test was 0.2 mm/s. The beads were subjected to a compressive force of up to 50%.

### 2.4. Cytotoxicity Assays

#### 2.4.1. Cell Culture

Normally established cell lines HaCaT (human keratinocytes) and BALB/c 3T3 (murine fibroblast) were used. The cells were grown in plastic flasks (25 cm^2^) with Roswell Park Memorial Institute (RPMI) 1640 medium, supplemented with 10% inactivated fetal bovine serum (FBS) and 1% antibiotic solution (penicillin and streptomycin). The cultures were incubated at 37 °C in an atmosphere containing 5% CO_2_. The medium was changed every 48 h, and when the culture reached confluence, the subculture was treated with trypsin-EDTA, until complete release of the cells. The released cells were transferred to a new plastic flask or 24-well plates.

#### 2.4.2. MTT Reduction Assay

HaCaT and BALB/c 3T3 cells were distributed in 24-well plates using a density of the 4 × 10^4^ cell/mL and 6 × 10^4^ cell/mL, respectively, and were incubated at 37 °C with 5% of CO_2_ for 24 h. Later, the cells were treated with different concentrations of SF, KGM and SF/KGM for 24 h. After incubation, the medium was removed from the cells and 1 mL of 3-(4,5-dimethylthiazol-2-yl)-2,5-diphenyltetrazolium bromide (MTT) solution (0.5 mg/mL in FBS free culture medium) was added to each well. After incubation for 2 h at 37 °C, the MTT solution was removed and the formazan crystal was solubilized in 1 mL of ethanol. The plate was shaken for 5 min and the absorbance of each well was read in the spectrophotometer (Infinity M200Pro, Tecan, Männedorf, Switzerland). The measured absorbance at λ = 570 nm was normalized to % of control [19]. This value was calculated by multiplying the absorbance of a treated well by 100 and dividing it by the average absorbance of control well, which was considered 100%.

### 2.5. Statistical Analysis

Each experiment was carried out in triplicate unless otherwise specified. All results are presented as the mean ± standard deviation (SD). The experimental data from all the studies were analyzed using Analysis of Variance (ANOVA). Statistical significance was set to *p*-value ≤ 0.05.

## 3. Results and Discussions

### 3.1. Size Beads and Surface Tension

**Figure 2:**
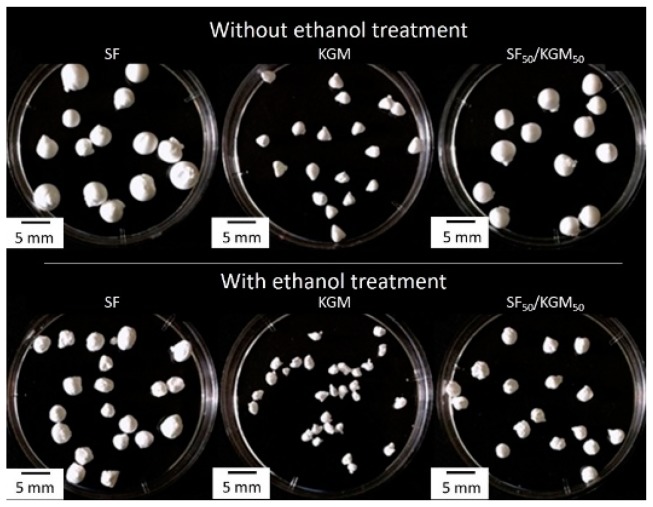
Images of the synthesized beads.

Figure 2 shows images of the beads treated without and with ethanol, and Table 1 presents the surface tension of solutions and diameters of beads. Beads under ethanol treatment showed smaller diameters than beads without treatment: SF, KGM and SF_50_/KGM_50_ presented a decrease of 8.9%, 7.6% and 23.4%, respectively. In the case of SF_50_/KGM_50_ beads, they showed an intermediate size between both pure natural polymers beads, due to the intermolecular interactions between both substances. According to the literature, higher surface tensions should produce higher diameters of drops, because the drop increases its size adding mass until the surface tension cannot keep it together [20]. However, in our case, the opposite was, solutions with higher surface tensions produced smaller diameter of beads (See Table 1 and Figure 2). These results suggest that other factors, in addition to the cohesive force between molecules, affect the diameter of the beads. An example is the molar mass of the natural polymer: higher molar mass would produce more compacted beads. In native form, the molar mass of SF is around 25 kDa for light chains and 325 kDa for heavy chain [21], while KGM is so much higher, around 1.50 × 10^6^ Da [22].

### 3.2. Morphological Characterization

**Figure 3:**
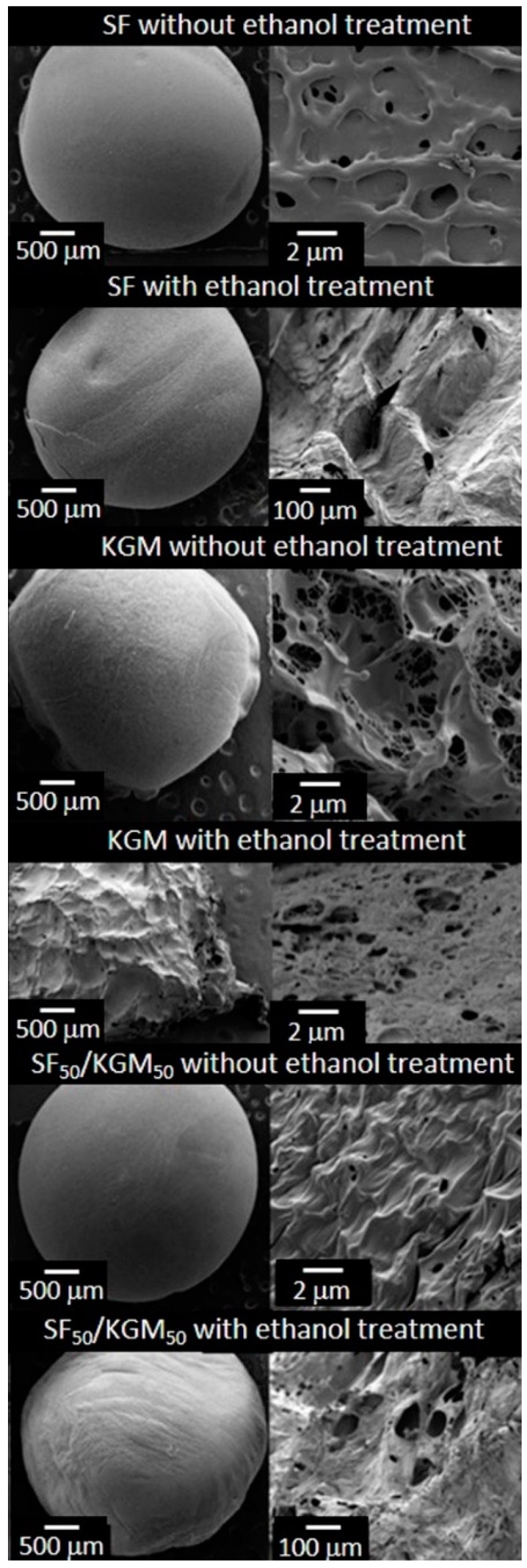
SEM images of the beads surface.

Figure 3 shows the SEM images of beads surface. All cases presented pores and rough surfaces. The fast freezing with liquid nitrogen and the freeze-drying in vacuum-process used during the syntheses, generated these particular characteristics. In the case of SF beads, it is possible to observe that both samples, without and with ethanol treatment, presented homogenous surfaces. However, the untreated samples exhibited pores of 0.97 ± 0.2 μm, and the treated samples presented pores of 76.0 ± 26.5 µm. The KGM beads were the most affected by the ethanol treatment. Samples without treatment showed a spongy, homogeneous surface and many pores with a size of 1.3 ± 0.3 μm. The treated beads presented a heterogeneous, texturized surface and higher pores with a size of 32.5 ± 7.0 μm. The untreated samples of SF_50_/KGM_50_ exhibited a rough and homogeneous surface with pores of 0.5 ± 0.2 μm. On the contrary, the treated beads presented large pores of 64.2 ± 13.4 μm. According to studies of Teimouri et al. (2015), the range of the pore size should be from 50 to 150 μm for successfully grown fibroblast cells and keratinocytes, since this size favors the transport of nutrients and oxygen [23]. In the case of the studies of Sepulveda et al. (2000), authors indicated that human osteoblasts could penetrate pores with a size between 100 and 200 μm, and a porosity above 30% is needed to facilitate oxygenation and nutrient transport [24]. Then, in the case of the synthesized beads, SF and SF_50_/KGM_50_ treated with ethanol should provide the requirements for the cellular growth of fibroblasts and keratinocytes.

### 3.3. Chemical Characterization

**Figure 4:**
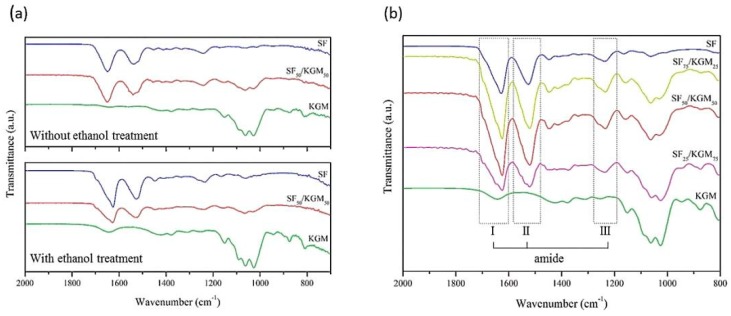
(**a**) FTIR-ATR spectra of beads, and (**b**) FTIR-ATR spectra of SF/KGM beads treated with ethanol obtained at different ratios of SF and KGM (*v*/*v*).

To chemically characterize the synthesized beads, FTIR-ATR analyses were performed (Figure 4). Figure 4a presents the spectra of the untreated and treated beads with ethanol. In the case of the untreated SF beads, amide bands corresponding to the silk I conformation (α-helix or random coil) were identified [25]: amide I (1638–1655 cm^−1^), amide II (1535–1540 cm^−1^) and amide III (1230–1240 cm^−1^). The amide bands I, II and III are attributed to C=O stretching, N–H deformation and O–C–N folding, respectively [25]. After the ethanol treatment the amide bands of the silk I were shifted to the amide bands corresponding to silk II (β-sheet): amide I (1616–1637 cm^−1^), amide II (1515–1530 cm^−1^) and amide III (1250–1260 cm^−1^), which is a more stable conformation of the SF [26]. Both KGM spectra, untreated and treated beads, presented their typical bands [27] in the range of 1000–1300 cm^−1^: at 1024 (C–O and C–OH), 1066 (C–H), 1153 (C–O), and 1247 cm^−1^ (CH_2_OH and C(=O)O). The main difference in the treated bead appears in the 1643 cm^-1^ band, which is attributed to the deformation in the plane of the water molecule due to crystallization. This is an intermolecular hydrogen bonding interaction originated by treatment in an ethanol and alkali agent [27]. Finally, a slight band at 1730 cm^−1^ (C=O) was faded, which is related to an increase of deacetylation, causing an increase of hydrogen bonds [28]. As expected for the SF_50_/KGM_50_ beads, previously discussed bands corresponding to SF and KFM were identified. In fact, as the case of SF, SF_50_/KGM_50_ spectra exhibited a shift of higher energies: from amides of silk I to amides of silk II. The ethanol treatment made a more stable conformation of beads. Yang et al. (2000) reported the same tendency for SF/cellulose films [29]. Also, a shift in the amide bands was observed when comparing the spectra of SF with SF_50_/KGM_50_, which is usually accompanied by the formation of hydrogen bonds between polymers [30].

To achieve a better understanding of the chemical conformation of the blend SF/KGM, FTIR-ATR analysis was also performed on the treated beads using different proportions of SF/KGM (*v*/*v*) (Figure 4b). As KGM is added to the blend, the conformational transition of amides from α-helix conformation and random coil to β-sheet is increased. Bands shift from 1650, 1540 and 1250 cm^−1^ to 1630, 1522 and 1230 cm^−1^. These results may indicate that a part of the random coil formation was converted to β-sheet with the addition of KGM, by intermolecular hydrogen bonds formed between the natural polymers [31]. According to the literature, when SF was mixed with other natural polymers containing hydroxyl groups (cellulose, alginate, heparin, and others), cross-linking between the polymers may benefit the conformational transition of SF [30]. Chen et al. (1997) performed structural analyzes on SF and chitosan films [32]. Authors found that SF exhibited β-sheet structure, indicating that hydrogen bonding was formed between SF and chitosan in the blend films. Also, they asserted that SF could use the rigid molecules of chitosan to extend SF molecules, resulting in the induction of the conformational β-sheet formation caused by hydrogen bonding. This study is consistent with the obtained results. Deconvolution corresponding to amide bands in FTIR-ATR spectra of SF/KGM was performed to analyze the evolution of β-sheet when KGM was added to the blend (Figure S1, Supplementary Materials). Table S1 (Supplementary Materials) shows the percent of β-sheet structure in each case of the amide. Results indicated that, as the proportion of KGM in the blend is increased, the percentage of β-sheet increase: 29.07%, 1.94% and 13.32% for amides I, II and III, respectively. These results show that part of the random coil was converted to β-sheet with the addition of KGM. The groups of KGM that can interact with SF are CH_2_OH, COOH and OH. If the CH_2_OH and COOH do not participate in the hydrogen bonding, the domain of the random coil conformation should be increased. However, as results show through the increasing of β-sheet, such groups may participate in the intermolecular bonds with the NH group of SF [33,34,35].

### 3.4. Thermal Characterization

**Figure 5:**
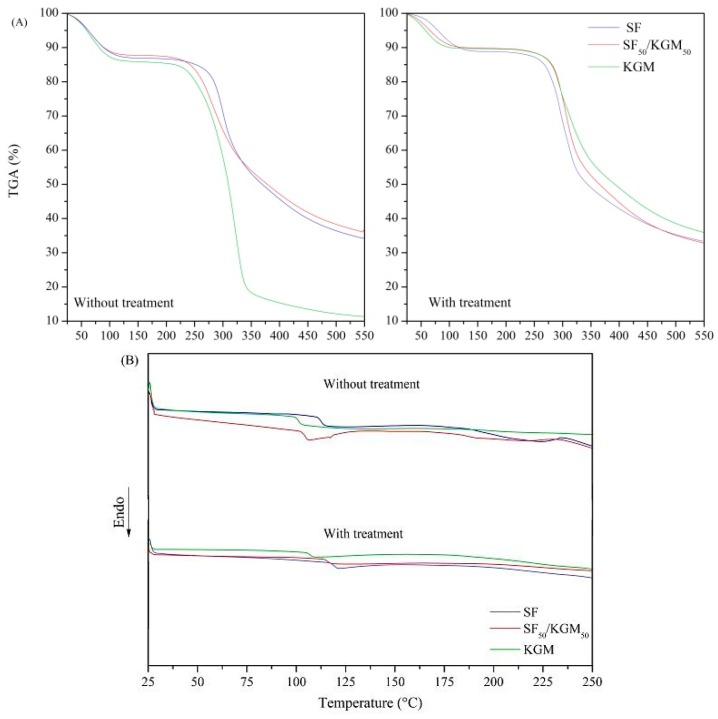
(**A**) TGA and (**B**) DSC analysis of beads.

Thermal stability of the beads was studied by thermogravimetric analysis (Figure 5A). The experiments involved three steps: (1) vaporization and removal of moisture in beads (to 100 °C), (2) material decomposition (at a different range for each case), and (3) thermal degradation and carbonization of the compounds (375 °C for all cases). In the case of SF samples, they presented a loss mass related to the breakdown of peptide bonds and side-chains of the residual amino acids [14,36]. Untreated and treated beads presented similar loss of mass in the second step: 36% and 33%, respectively. However, the decomposition temperature range of the beads treated with ethanol (235–297 °C) was higher than the untreated beads (216–283 °C). This suggests that ethanol post-treatment provides thermal stability, possibly due to the structural transition from silk I to silk II, which is more stable. The loss of mass of KGM is related to the decomposition of saccharide rings and the disintegration of the molecule chains [37]. In the second step, untreated beads showed 84% of loss mass, and treated beads just reached 55% of losing mass. The decomposition temperature range of untreated and treated beads were 223–322 °C and 260–296 °C, respectively. Although the maximum decomposition temperature was lower in treated samples than untreated samples, the initial temperature of decomposition was increased due to the higher deacetylation [38]. As ethanol decreases the acetyl groups of KGM, the intermolecular interactions are strengthened, and this consequently improves the thermal stability of the material [39]. In the case of SF_50_/KGM_50_ beads, the loss of mass was very similar between untreated and treated samples: 43% and 46%, respectively. Regarding the decomposition temperatures ranges, both cases were similar but with a slight increase in the maximum decomposition temperature: 233–299 °C for untreated beads, and 233–303 °C for treated beads.

The interaction between the polymers can also be analyzed by the glass transition temperature (*T*_g_) of the beads, which were obtained from the DSC (Figure 5B). The calculated *T*_g_ was 116, 101 and 104 °C for the untreated samples of SF, KGM and SF_50_/KGM_50_, respectively. These results confirm that SF_50_/KGM_50_ was a miscible mixture because its *T*_g_ is an intermediate value between both *T*_g_ of pure polymers [40]. The same tendency was observed in the treated samples: 119, 107, and 108 °C for SF, KGM, and SF_50_/KGM_50_ beads, respectively. One point to highlight is that *T*_g_ of treated samples was higher than untreated ones, confirming that the ethanol treatment enhanced the stability of beads.

### 3.5. Crystallinity

**Figure 6:**
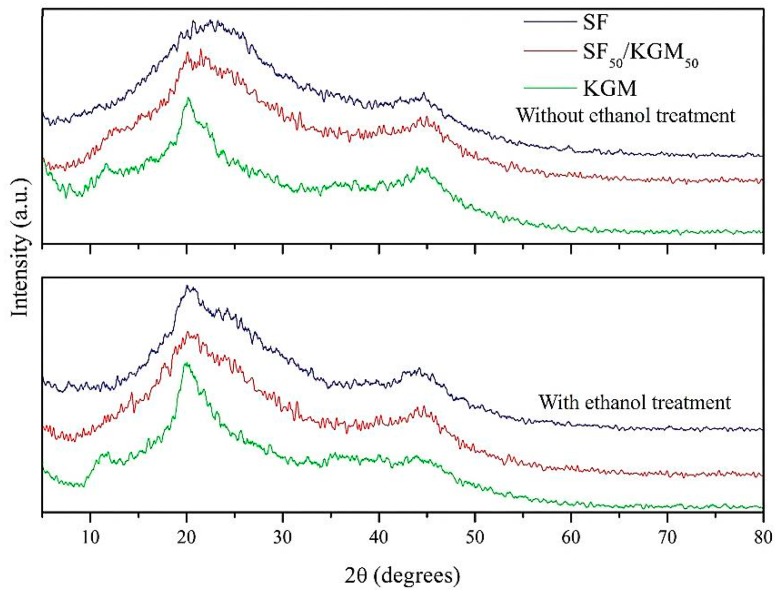
XRD diagrams of beads.

Figure 6 shows the X-ray diffractograms of samples. In the case of SF, values of 2θ at 23° and 45° are attributed to silk I (α-helix) while values at 21° are attributed to silk II (β-sheet) conformation. After ethanol treatment, the matrix becomes more crystalline in the silk II (β-sheet) structure, which shows that the protein chains of SF are more organized and energetically stable when they are treated with solvents or temperature [14,25,41]. According to the previous study of KGM cristallinaty [28], KGM beads presented their typical peak around 2θ = 20°. However, the increment of the peak around 12°, after the ethanol treatment, exhibited a higher deacetylation degree produced by the solvent [28]. As SF_50_/KGM_50_ is a blend, their diffractograms presented lower crystallinity than diffractograms of pure SF and KGM [42]. However, in this case, significant changes in crystallinity were not caused by the ethanol treatment, as the cases of pure polymers.

### 3.6. Mechanical Assays

**Figure 7:**
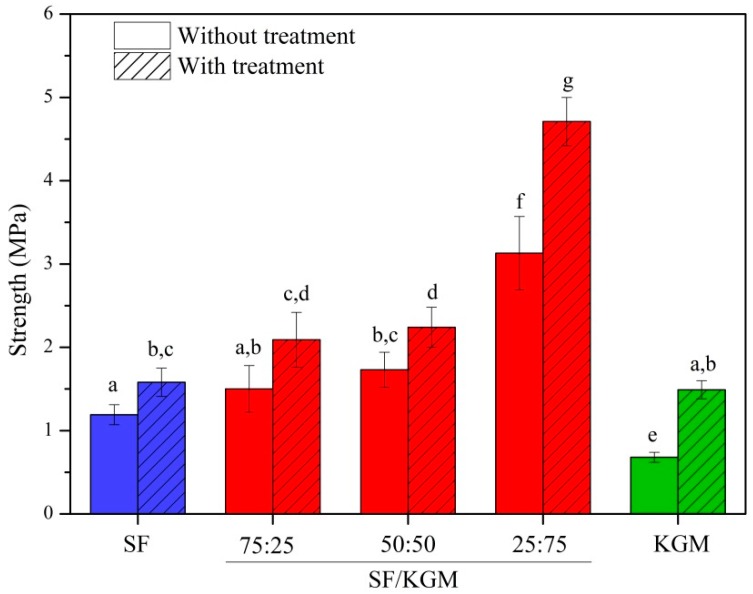
Mechanical properties of beads obtained at different ratios of SF and KGM (*v*/*v*). Mean ± SD (*n* = 15), means with the same letter indicate that there is no significant difference (*p* < 0.05) by the Tukey test.

Figure 7 presents the mechanical tests applied to samples obtained at different ratios of SF and KGM (*v*/*v*). For all cases (pure polymer beads and different blends) the ethanol treatment significantly improved the strength of samples. Regarding the SF component, the organic solvent dehydrated its chains favoring hydrogen bonds and Van der Waals forces. It, thus, changes to a more stable structural conformation [3]. According to the literature [16,43], the increasing of the mechanical resistance is caused by higher amounts of β-sheet structure, which is more stable. In the case of KGM, ethanol assisted in the deacetylation of the molecule [16], facilitating the formation of hydrogen bonds and hydrophobic interactions between chains composing a gel network structure [7,42]. This result was consistent with those obtained by ATR-FTIR, TGA and XDR analyses. When KGM was incorporated into SF, the mechanical strength of beads was increased. This is strongly related to intermolecular interactions of hydrogen bonds formed between both polymers [30,31,44], providing a more stable structure than pure polymer beads.

Park et al. (1999) found that the mechanical tensile strength of SF/CHI membranes depended on CHI content. In fact, blends presented higher resistance than pure polymers, possibly due to the increase in the conformational transition β-sheet [9]. Other works in the literature have reported the increase of strength when one of the components is slightly added. For example, Yin et al. (2012) reported that scaffolds of collagen:chitosan:P(LLACL) were stronger when the ratio was 20:5:75 in comparison to pure P(LLACL) and collagen-chitosan blended scaffold (80:20:0) [45].

### 3.7. Cytotoxicity Assays

**Figure 8:**
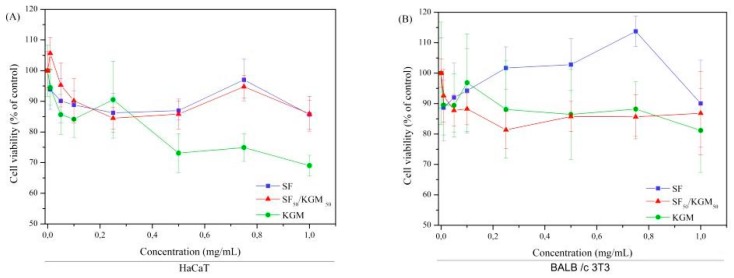
Cytotoxicity of solutions to (**A**) HaCaT and (**B**) BALB/c 3T3 cells. Mean ± SD (*n* = 3).

**Figure 9:**
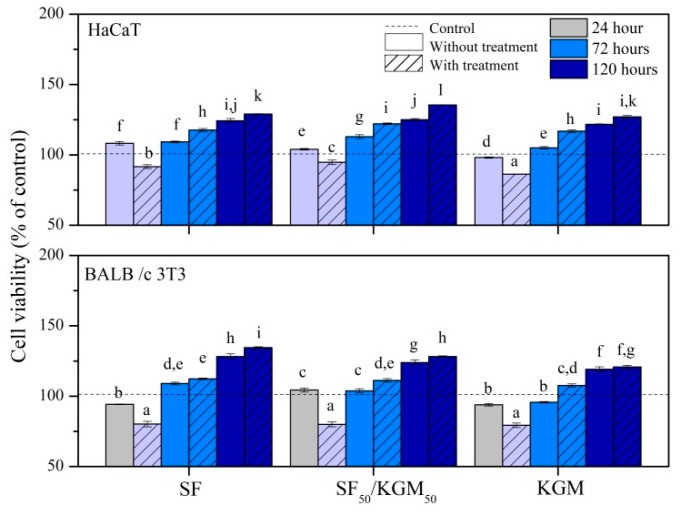
Cytotoxicity of beads to HaCaT and BALB/c 3 T3 cells. Mean ± SD (*n* = 3), means with the same letter indicate that there is no significant difference (*p* < 0.05) by the Tukey test.

The cytotoxicity of solutions (Figure 8) and beads (Figure 9) was tested with two kinds of cell lines: human keratinocytes (HaCaT) and murine fibroblast (BALB/c 3T3).

Figure 8 shows that the natural polymers did not present cytotoxicity up to of 1 mg/mL. The IC50 value (reduction of 50% of the cell population) was not found in any of lineages. Also, it is noted the presence of SF in the SF/KGM solution favored the cytotoxicity of the SF since the profiles were very similar.

KGM showed a tendency towards low cytotoxic profile for HaCaT in the concentration of 1 mg/mL since it is within the limit of the recommended in ISO 10993-5 (2009) [46]. Based on the MTT tests, it was not possible to estimate how the polymer acted in the cells, so it is necessary to perform signaling pathways, apoptosis (programmed cell death) and inflammatory process to verify the mechanism of decay of the cytotoxic profile.

In general, the decrease in cytotoxicity at the highest concentration tested was approximately 30% for HaCaT and 20% for BALB/c 3T3. When the cells were exposed to the combination of both natural polymers, it did not show cytotoxicity to the studied strains when compared to MTT reduction. Thus, the obtained beads could be applied in the field of tissue engineering.

Regarding the beads (Figure 9), in general, untreated and treated beads did not present cytotoxicity for both kinds of cell lines, since the cell viability for all cases and times was higher than 50%. Materials are considered cytotoxic only if it presents <50% of viable cells at any time [46,47]. However, during first 24 h of cell culture, treated beads showed a slight reduction of cell viability. This could be due to some residual traces of ethanol. Concentrations of this solvent above 0.1% may already be considered toxic to cells: dividing cells, trypsinization, and apoptosis. At 72 and 120 h of culture, all samples showed higher cell proliferation than control, probably due to natural selection occurring during the first 24 h. Nevertheless, the treated beads exhibited better cell viability than untreated samples. After 72 h, this increase can lead it to be assumed that treated beads acted as a dose-response system. Although in some cases, cell viability was similar to pure polymers, treated samples of SF_50_/KGM_50_ reached the highest values of cell viability: 136% ± 0.1% for HaCaT, and 135% ± 1% for BALBc/3T3, both at 120 h.

## 4. Conclusions

SF, KGM, and SF/KGM beads were obtained by a viable protocol that produced porous beads by dripping into liquid nitrogen, with a post-treatment using ethanol. Ethanol treatment reduced the diameter of beads but significantly increased the size of surface pores; making them viable for cell adhesion and growth. Physicochemical analysis of samples showed that SF/KGM beads were integrated due to the formation of hydrogen bonds between both polymers. Also, beads treated with ethanol exhibited more crystallinity and thermal stability, which were generated by a conformational transition from silk I (α-helix or random coil) to silk II (β-sheet) in SF, and an increase in the deacetylation of KGM. Due to these chemical changes, treated SF/KGM beads showed higher mechanical resistance than pure SF and KGM beads under compression tests. Finally, all beads did no present cytotoxicity effect for HaCaT and BALBc/3T3 cells up to 120 h and treated SF/KGM beads reached the highest values of cell viability. In general, the obtained porous blend beads presented improved characteristics compared to pure polymer beads, which could be used in the field of biomedicine.

## Figures and Tables

**Table 1:** The surface tension of SF, KGM, and SF_50_/KGM_50_ solutions and diameters of SF, KGM, and SF_50_/KGM_50_ beads.

Samples	Surface Tension of Solutions (mPa) *	The Diameter of Beads without Ethanol Treatment (mm) *	The Diameter of Beads with Ethanol Treatment (mm) *
SF	45.2 ± 0.2 ^a^	3.4 ± 0.1 ^d^	3.1 ± 0.1 ^h^
KGM	63.7 ± 0.8 ^b^	2.0 ± 0.2 ^f^	1.8 ± 0.1 ^e^
SF_50_/KGM_50_	50.7 ± 0.7 ^c^	3.2 ± 0.15 ^d^	2.5 ± 0.1 ^g^

* Mean ± SD (*n* = 3), mean with the same letter indicate that there is no significant difference (*p* < 0.05) by the Tukey test.

## Supplementary Materials

The following are available online at http://www.mdpi.com/2073-4360/10/8/923/s1, Figure S1: Deconvolution of FTIR-ATR spectra of SF/KGM beads treated with ethanol obtained at different ratios of SF and KGM (*v*/*v*), Table S1: β-sheet percent obtained from the deconvolution of FTIR-ATR spectra of SF/KGM beads treated with ethanol obtained at different ratios of SF and KGM (*v*/*v*) for the amide I, II and III. In red the percentage referring to the conformation β-sheet.
