# Supplementary material for: Synthesis and Properties of Silk Fibroin/Konjac Glucomannan Blend Beads
Source: Polymers (Basel). 2018 Aug 18;10(8):923. doi: 10.3390/polym10080923 (PMC6403648; doi:10.3390/polym10080923)
Supplement: Supplementary file 1 [file polymers-10-00923-s001.pdf]

Supplementary Information

Synthesis and Properties of Silk Fibroin/Konjac Glucomannan Blend Beads

Carla Giometti França, Vicente Franco Nascimento, Jacobo Hernandez-Montelongo, Daisy Machado, Marcelo Lancellotti and Marisa Masumi Beppu

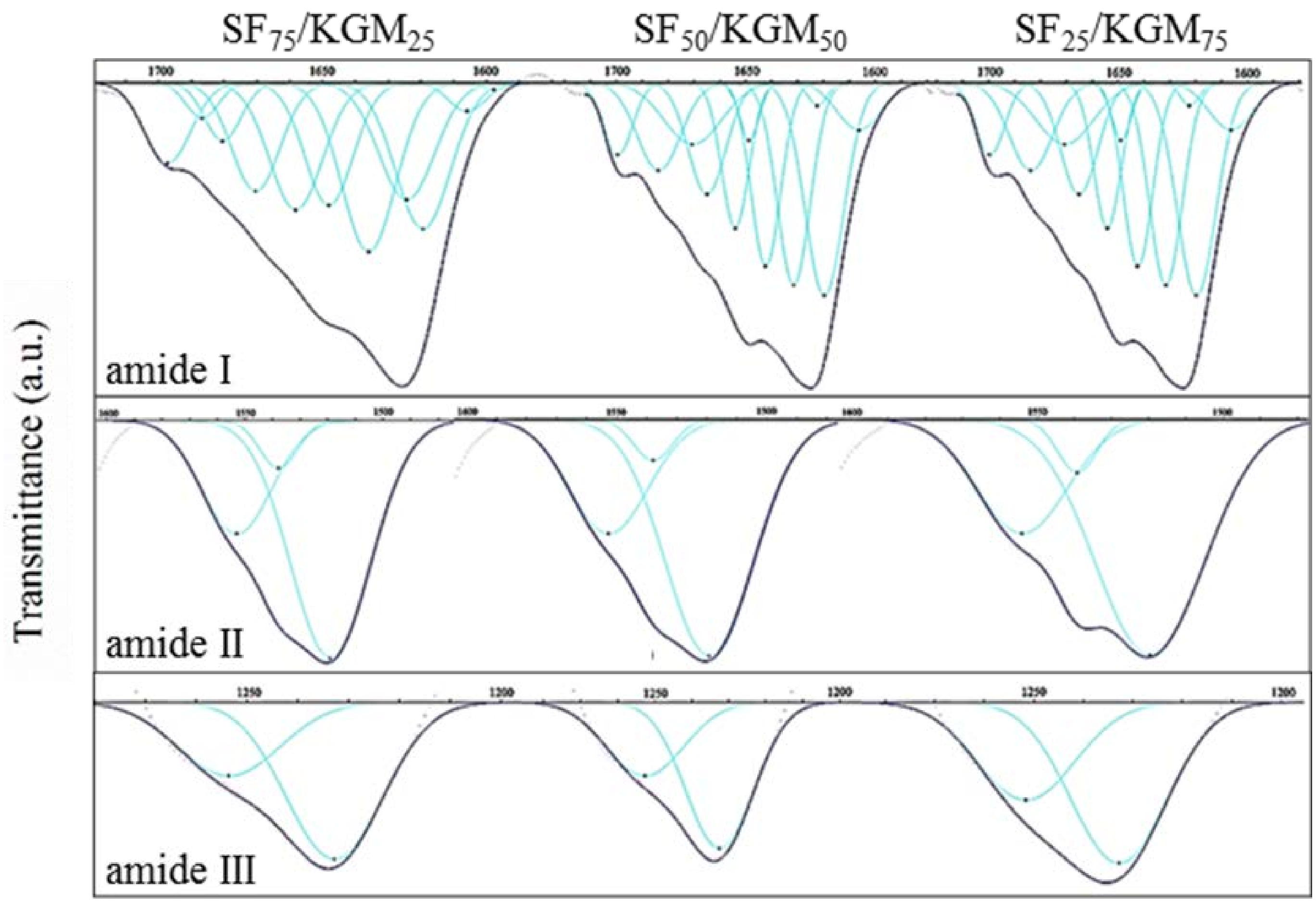

Figure S1. Deconvolution of FTIR-ATR spectra of SF/KGM beads treated with ethanol obtained at different ratios of SF and KGM (v/v).

Table S1. β-sheet percent obtained from the deconvolution of FTIR-ATR spectra of SF/KGM beads treated with ethanol obtained at different ratios of SF and KGM (v/v) for the amide I, II and III. In red the percentage referring to the conformation β-sheet.

		Amide I						Amide II			Amide III	
**SF_{25}/KGM_{75}**	Wavenumber (cm^{-1})	1599	1622	1650	1668	1682	1699	1519	1539	1553	1232	1253
	Area (%)	1.27	34.12	45.42	2.15	11.70	5.33	67.38	5.43	27.18	61.71	38.29
	β-sheet (%)	39.45						67.38			38.29	
**SF_{50}/KGM_{50}**	Wavenumber (cm^{-1})	1601	1622	1647	1683	1668	1699	1519	1538	1552	1232	1252
	Area (%)	1.21	23.86	61.17	8.46	1.06	4.25	66.30	5.08	28.62	63.51	36.49
	β-sheet (%)	28.10						66.30			36.50	
**SF_{75}/KGM_{25}**	Wavenumber (cm^{-1})	1601	1622	1647	1668	1683	1699	1519	1538	1553	1232	1251
	Area (%)	1.11	23.33	61.54	1.09	8.28	4.65	66.08	6.29	27.62	66.79	33.21
	β-sheet (%)	27.98						66.08			33.2	
